# Water-Insoluble Films from Upcycled Babassu Coconut Byproducts: An Alternative Material for Single-Use Oil Sachets

**DOI:** 10.1021/acsomega.5c07743

**Published:** 2025-12-29

**Authors:** Letícia de Oliveira Gonçalves, Patrícia Marques De Farias, Yan Fonseca dos Santos, Jefferson Santos de Gois, Bianca Chieregato Maniglia, Ana Elizabeth Cavalcante Fai

**Affiliations:** † Food and Nutrition Graduate Program, Federal University of the State of Rio de Janeiro—UNIRIO, Av. Pasteur, 296, Urca, Rio de Janeiro/RJ 22290-240, Brazil; ‡ Sustainable Packaging Institute SPI, Faculty of Life Sciences, Albstadt-Sigmaringen University, Anton-Günther-Str. 51, Sigmaringen 72488, Germany; § Laboratory of Multidisciplinary Practices for Sustainability (LAMPS), Institute of Nutrition, of Rio de Janeiro State University (UERJ), R. São Francisco Xavier, 524, Maracanã, Rio de Janeiro/RJ 20550-900, Brazil; ∥ Department of Analytical Chemistry, Rio de Janeiro State University (UERJ), R. São Francisco Xavier, 524, Maracanã, Rio de Janeiro/RJ 20550-900, Brazil; ⊥ São Carlos Institute of Chemistry, 28133 University of Sao Paulo (USP), Av Trabalhador São Carlense, São Carlos/SP 13566-590, Brazil

## Abstract

This research presents the development of an innovative water-insoluble film intended for use as an oil sachet. A polymeric matrix composed of babassu mesocarp flour, or its blend with babassu cake supernatant, was prepared at both native pH and pH 12. The alkaline treatment combined with supernatant (BF-BCFS12) significantly enhanced the crystallinity index (reducing from 7.35% to 3.21%), heat resistance (increasing from 57.56 to 71.25 °C), solubility (increasing from 0.14% to 1.23%), and luminosity (decreasing from 33.86 to 20.43) in comparison to the pure mesocarp film. An innovative permeability evaluation conducted using cream crackers indicated that the alkali-treated films preserved texture more effectively than the original packaging. The antiultraviolet property emerged as a critical factor in selecting BF-BCFS12 as a sachet for soybean and olive oils stored for 6 days under conditions conducive to oxidation (50 ± 2 °C under light at a distance of 6 cm). The surface wettability of BF-BCFS12 exhibited moderate hydrophilicity in water (approximately 60°) and lipophilicity in oil (less than 17°). The sachet demonstrated an initial strength of 83.32 N, and after 6 days, the peroxide value of soybean oil was found to be 1.45 times higher, while that of olive oil was 2.8 times lower than that observed in the original package, with acidity and color differences remaining unchanged. To our knowledge, this is the first study to incorporate babassu cake into a polymer blend film.

## Graphical abstract

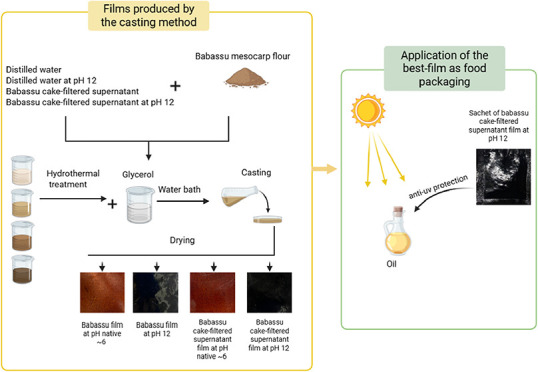

## 1 Introduction

Babassu (*Attalea speciosa*), native to northern and northeastern Brazil,^1^ is the country’s most important oil palm for plant extractivism.^2^ In 2022, approximately 57,000 tons of oil were produced annually,^3^ generating a significant amount of waste known as babassu cake, which is either discarded or utilized as animal feed.^4^

Babassu cake has potential as a polymer matrix or reinforcing agent in the development of biobased films due to its carbohydrate content (41.5%), protein content (18.8%), and lipid content (28.8%).^5,6^ A coproduct of babassu, its mesocarp, is composed of starch (84.57%), dietary fiber (11.11%), proteins (1.77%), lipids (1.51%), and phenolic compounds (8.3 mg GAE), and it can form films.^7^ In this context, alkaline treatment can enhance the extraction and incorporation of micro- and macromolecules and improve film properties.^7–9^

Petroleum-based plastics are predominant due to their low cost, lightweight, flexibility, and durability.^10^ However, their long-term environmental impact is incompatible with the short shelf life of food, highlighting the need for sustainable, biologically sourced alternatives.^11^

The innovation of this article lies in its pioneering research on using supernatant babassu cake to develop food packaging. By upcycling an abundant byproduct into a high-value material, this approach advances circular bioeconomy goals and provides credible socio-environmental cobenefits throughout the babassu supply chain. The aim of this study was to develop a flexible film using babassu mesocarp flour or a blend with babassu cake supernatant under native pH and alkaline conditions. The study assessed the mechanical, physical, optical, thermal, and chemical properties, verifying the effects of the cake supernatant and alkaline treatment compared to the mesocarp film at native pH. The most effective film was tested for its ability to retard lipid oxidation when employed as packaging for oils.

## 2 Materials and Methods

### 2.1 Materials

The babassu mesocarp flour (BM) was provided by Vem do Xingu (Altamira, PA, Brazil), and the babassu cake was supplied by Florestas Brasileiras Ltd.a (Itapecuru Mirim, MA, Brazil). Glycerol was obtained from Chepplier (Rio de Janeiro, Brazil), and NaOH from Dinâmica Química Contemporânea Ltd.a (São Paulo, Brazil). Soybean oil and organic extra virgin olive oil were purchased at a local market in Rio de Janeiro.

### 2.2 Characterization of Babassu Cake: Centesimal Composition, Colorimetric Parameters, and Multielement Analysis

The babassu cake, which was unsieved (BC) and sieved to 100 mesh (BC100), was characterized based on its centesimal composition as defined by the Adolf Lutz Institute (2008) [1]. Color coordinates (Lab*) were measured using a colorimeter (3nh Y53020, China), and chroma (C*) was calculated as described by Fai et al. [2]. Multielement analysis was performed using ICP-OES [3].

### 2.3 Preparation of Film-Forming Suspensions Using Hydrothermal Treatment and Film Development by Casting

BM was used as the polymeric matrix for the film-forming suspensions (FFS) following Maniglia et al.^7^ For each FFS, 4 g of BM was suspended in 96 g of BCFS (pH ∼ 6 or 12) and hydrothermally treated in an autoclave at 121 °C for 30 min^12^ (Phoenix AV50 n 10001, Brazil). Glycerol (30 g/100 g BM) was added as a plasticizer. Films were designated BF-BCFS (pH ∼ 6) and BF-BCFS12 (pH 12). To evaluate the effect of BCFS, two additional FFS were prepared: BM with water at pH ∼ 5 (BF) and at pH 12 (BF12). All FFS (Table 1) were cast on acrylic plates (0.28 g/cm^2^) and dried at 35 °C for 18 h (Marconi, Brazil). Films were conditioned in a desiccator (25 °C, 53% RH) for 48 h prior to analysis. BF served as the control.

**Table 1:** Film-Forming Suspensions Developed Based on Babassu Mesocarp and Cake^a^

		film-forming suspensions				
film identification		BM (g)	distilled water (g)	BCFS (g)	pH	glycerol (g)
1	BF	4	96	-	∼ 6	1.2
2	BF12	4	96	-	12	1.2
3	BF-BCFS	4	-	96	∼ 6	1.2
4	BF-BCFS12	4	-	96	12	1.2

a BM: babassu mesocarp flour; BCFS: babassu cake-filtered supernatant; BF: babassu-based film; BF12: babassu-based film at pH 12; BF-BCFS: babassu cake-filtered supernatant film; BF-BCFS12: babassu cake-filtered supernatant film at pH 12.

### 2.4 Characterization of Babassu-Based Films

#### 2.4.1 Thickness and Mechanical Properties

Film thickness was measured with a 0–25 mm digital micrometer (Digimess, Brazil) as described by Andrade et al.^13^ Mechanical properties were assessed according to ASTM D882–12 ^14^ using quintuplicate samples in a TX-700 texturometer (Lamy Rheology, France).^15^

#### 2.4.2 Moisture Content and Solubility in Water

Moisture content was determined by drying the films until constant weight.^16^ Water solubility was determined by the difference between the final and initial mass of the film discs.^15^

#### 2.4.3 Water Vapor Permeability by Permeation Cell and Analysis of the Texture of Cream Cracker Biscuits

Water vapor permeability (WVP) was measured using a modified ASTM E96/E96 M method^17^ by sealing films over permeation cells with silica gel. The barrier performance of the films was evaluated by analyzing the texture of cream cracker cookies as a complementary method to standard WVP testing. The biscuits, known for their sensitivity to moisture, were packaged in their original material or with the developed films (BF, BF12, BF-BCFS, BF-BCFS12) and then vacuum sealed for five seconds (RG-300L, Registron, Brazil). Texture was measured using a texturometer with a 10 mm cylindrical probe, a 100 N load cell, and 50% strain.^18^ Samples were stored at 20 ± 5 °C and 50 ± 5% relative humidity and analyzed on days 0, 3, 7, and 15 according to Lopes et al.^19^ with modifications.

#### 2.4.4 Colorimetric Characterization, Light Transmission, Opacity, Visual Aspect, and Microstructure

Film color and total color difference (ΔE*) were obtained by averaging five surface measurements according to Fai et al.,^20^ using a colorimeter (3nh Y53020, China). Light transmittance (200–800 nm) was measured with a UV–Vis spectrophotometer (Biochrom Libra S22, U.K.), and opacity was calculated as absorbance at 600 nm divided by film thickness (mm).^21^ Visual appearance was captured with an iPhone 11 (Apple; 4000 × 3000 pixels). Film morphology (vertical and transverse sections) was examined by scanning electron microscopy (SEM, MEV-LEO 440), where samples were mounted on double-sided carbon tape, gold-coated by cathodic spraying, and imaged at 15 kV and 1000×.

#### 2.4.5 Chemical and Thermal Properties

Film chemical structure was analyzed by Attenuated Total Reflectance Fourier Transform Infrared Spectroscopy (ATR–FTIR, IR-Affinity 1, Shimadzu, Japan) over 4000–650 cm^–1^ (4 cm^–1^ resolution, 32 scans). Thermal properties were evaluated by Differential Scanning Calorimetry (DSC, TA 2010, TA Instruments) with cryoscopic cooling according to De Farias et al.,^22^ in which 5 mg dry samples were sealed in aluminum pans and scanned under N_2_ (45 mL min^–1^) from −150 to 150 °C at 10 °C min^–1^. The data were processed in Universal Analysis 2000 (TA Instruments). Thermogravimetric Analysis (TGA) was performed on 10 mg samples using a TGA-Q500 (TA Instruments) in platinum pans, heated from 10 to 700 °C at 10 °C min^–1^ under N_2_, recording continuous mass changes.^6^

#### 2.4.6 Crystallinity Index

The crystallinity index of the films was measured using an X-ray diffractometer (Siemens model D5005, Germany) at 40 kV and 30 mA, utilizing copper Kα radiation and a monochromatic filter at room temperature.^22^

#### 2.4.7 Surface Wettability

Surface wettability in water and olive oil was evaluated through contact angle measurements using an OCA-20 device (Dataphysics, Germany).^7^

#### 2.4.8 Heat Seal Strength

Two 45 × 10 mm film pieces were overlapped and heat sealed at approximately 70 °C for 5 s using a vacuum sealer (RG-300L, Registron, Brazil). The sealed samples were conditioned in triplicate for at least 48 h at 53% relative humidity (RH), and the seal strength was measured using a texturometer, following the methodology outlined by Alves et al.^23^

### 2.5 Film Application as Food Packaging

#### 2.5.1 Analysis of Soybean Oil and Extra Virgin Olive Oil in Sachets During Storage

Two 5.5 × 6.5 cm pieces of film were sealed to form sachets containing 4 g of soybean or olive oil. Sachets were subjected to accelerated oxidation for 6 days at 50 ± 2 °C under light (at a distance of 6 cm), according to the adapted methodology of Dong et al.^24^ The oils were analyzed for acidity, peroxide, and color difference indices.^20,25^ The oils in their original packaging, maintained under the same conditions, served as controls. All samples were analyzed in duplicate on days 0, 3, and 6.

#### 2.5.2 Evaluation of Sachet Stability During Storage

The stability of the sachets was evaluated in triplicate on days 0, 3, and 6, examining color (ΔE*), thickness, and maximum strength using the methods described in sections 2.4.1 and 2.4.4.

### 2.6 Statistical Analysis

Data were analyzed using one-way ANOVA (95% confidence) with Statistica 7.0. Tukey’s test was employed to identify significant differences (*p* <  0.05).

## 3 Results and Discussion

### 3.1 Characterization of Babassu-Based Films

#### 3.1.1 Thickness and Mechanical Properties

Table 2 presents the thickness and mechanical properties of the films. BF-BCFS and BF-BCFS12 films were 1.5 times thicker than BF and BF12, likely due to the fibrous texture and higher protein, carbohydrate, and lipid content of the BCFS additive.^26,27^ The increased fiber content enhances the thickness and porosity of BCFS-based films, as demonstrated by SEM. Meanwhile, the lipid fraction reduces surface hydrophilicity, a desirable feature for food packaging. However, the higher porosity may increase WVP, potentially affecting the film’s barrier efficiency.

**Table 2:** Mechanical, Physical, and Colorimetric Parameters of Films^a^

	mechanical properties				physical parameters			colorimetric parameters					
films	thickness (mm)	tensile strength at rupture (TS) (MPa)	elongation at break (EB) (%)	Young’s modulus (YM) (MPa)	moisture (%)	water solubility (%)	WVP (10^–10^ g.s^–1^.m^–1^.Pa^–1^)	L*	a*	b*	chroma value (C*)	Δ*E*	opacity (%)
BF	0.14 ± 0.01^c^	1.95 ± 0.25^a^	15.43 ± 1.54^c^	6.50 ± 1.49^b^	17.54 ± 4.72^a^	0.14 ± 0.08^b^	3.08 ± 1.44^a^	33.86 ± 2.15^a^	34.9 ± 1.13^a^	27.56 ± 0.96^a^	44.48 ± 1.30^a^	control	5.18 ± 1.02^a^
BF12	0.12 ± 0.01^d^	0.59 ± 0.23^b^	38.28 ± 5.99^a^	0.43 ± 0.12^c^	22.39 ± 5.86^a^	0.70 ± 0.30^a^	3.28 ± 1.05^a^	19.38 ± 1.70^c^	–0.15 ± 0.10^c^	0.42 ± 0.10^c^	0.46 ± 0.10^c^	46.74 ± 0.90^a^	NC^b^
BF-BCFS	0.20 ± 0.02^a^	1.67 ± 0.53^a^	9.56 ± 2.82^c^	13.20 ± 2.50^a^	11.71 ± 3.09^a^	1.07 ± 0.16^a,b^	3.61 ± 2.12^a^	28.26 ± 1.57^b^	28.77 ± 2.25^b^	19.05 ± 0.26^b^	34.56 ± 3.70^b^	12.19 ± 3.72^b^	7.33 ± 0.42^b^
BF-BCFS12	0.18 ± 0.02^b^	2.20 ± 0.14^a^	26.37 ± 2.28^b^	4.29 ± 0.32^b^	16.07 ± 3.32^a^	1.23 ± 0.27^a^	3.99 ± 1.38^a^	20.43 ± 1.80^c^	–0.09 ± 0.08^c^	0.41 ± 0.12^c^	0.32 ± 0.16^c^	46.54 ± 1.56^a^	NC^b^

a BF: babassu-based film; BF12: babassu-based film at pH 12; BF-BCFS: babassu cake-filtered supernatant film; BF-BCFS12: babassu cake-filtered supernatant film at pH 12. Values with different letters in the same column are significantly different according to Tukey’s test (*p* < 0.05). WVP: Water vapor permeability.

b NC: Not calculated. Absorbance at the limit set by the instrument (>+3). Transmittance could not be used in the formula due to lack of specific numerical data.

In contrast to this study, where all films exhibited a thickness greater than 0.12 mm, the thickness of babassu mesocarp films with alginate reported by Lopes et al.^28^ averaged 0.08 mm with the same suspension volume per cm^2^. This discrepancy may be attributed to the presence of glycerol and lipids, as well as their chemical interactions, which enhance moisture retention or form additional layers, thereby increasing thickness and weight.^29,8,30^

Among the films, BF12 exhibited the lowest tensile strength (0.59 ± 0.23 MPa), approximately three times lower than the others. However, it demonstrated the highest elongation at break (38.28 ± 5.99%), resulting in greater flexibility and lower brittleness (0.43 ± 0.12 MPa). This reflects the inverse relationship between strength and elongation.^31^ BF12 was followed by BF-BCFS12 (elongation at break (EB): 26.37 ± 2.28%; Young’s modulus (YM): 4.29 ± 0.32 MPa) and BF (EB: 15.43 ± 1.54%; YM: 6.50 ± 1.49 MPa).

The disruption of polysaccharide bonds and the introduction of plasticizers weaken the intramolecular interactions of starch, facilitating hydrogen bonding with plasticizers, which increases the mobility and flexibility of the chains.^31^ Films composed exclusively of babassu mesocarp (BM) contain more free sugars, enhancing these interactions. Alkaline treatment also improves elasticity by weakening hydrogen bonds; however, higher fiber content can reduce elasticity.^31^ The greater elongation of BF12 and BF-BCFS12 compared to the native pH films may be attributed to alkaline treatment, which enhances the availability of lipid and sugar plasticizers,^22^ as evidenced by FT-IR analysis.

Although BF-BCFS12 underwent the same alkaline treatment as BF12, it exhibited lower elongation, likely due to the higher fiber content in BCFS, which increases matrix rigidity and porosity, as evidenced by SEM. Despite showing lower tensile strength than the mesocarp films reported by Maniglia et al.^7^ (12.50 ± 1.23 MPa), all four films demonstrated greater elasticity (over 2.85% ± 0.16).

A tensile strength (TS) of approximately 2.6 MPa was observed, lower than the findings of Maniglia et al.^7^ In contrast, Lopes et al.^28^ indicated that the Young’s modulus (YM) remained unchanged at 24.38 MPa; however, a significantly lower YM of approximately 6 MPa was obtained, which is approximately four times lower than the previously reported value.

#### 3.3.2 Moisture Content and Solubility in Water

The moisture content of BCFS reported by Maniglia et al.^7^ was 11.82 ± 0.23%, whereas the present study observed a value of approximately 16.92 ± 4.24%. Solubility results (Table 3) show no significant difference for BF-BCFS (1.07 ± 0.16%), whereas BF12 (0.70 ± 0.30%) and BF-BCFS12 (1.23 ± 0.27%) exhibited a notable increase compared to the control (0.14 ± 0.08%). The higher solubility of BF-BCFS12 may be attributed to its protein content and the solubilization of lipids induced by alkaline treatment,^7,32,6^ a trend also reported by De Farias et al.^6^

**Table 3:** Texture Profile Analysis of Cream Crackers Packed in the BF, BF12, BF-BCFS, and BF-BCFS12 Films and Original Packaging^a^

	hardness (N)			strength maximum (N)			cohesiveness (N)		
day									
	3	7	15	3	7	15	3	7	15
BF	34.21 ± 2.17^a,A^	18.27 ± 2.50^c,d,B^	15.59 ± 2.44^b,B^	25.12 ± 0.58^a,A^	13.12 ± 2.36^c,B^	11.122.05^b,B^	0.16 ± 0.02^a,A^	0.15 ± 0.02^a,b,A^	0.17 ± 0.03^a,b,A^
BF12	33.06 ± 9.30^a,A^	29.32 ± 1.81^a,b,A^	25.97 ± 2.54^a,b,A^	24.54 ± 6.58^a,A^	22.53 ± 1.39^a,b,A^	18.58 ± 2.88^a,b,A^	0.18 ± 0.02^a,A^	0.18 ± 0.02^a,A^	0.19 ± 0.02^a,A^
BF- BCFS	29.27 ± 4.79^a,A^	22,67 ± 1.33^b,d,A^	22.62 ± 2.69^b,A^	20.47 ± 5.83^a,A^	14.09 ± 1.24^b,c,A^	17.76 ± 2.55^b,A^	0.18 ± 0.05^a,A^	0.12 ± 0.02^a,d,A^	0.19 ± 0.01^a,A^
BF-BCFS12	26.68 ± 0.49^a,A^	23.56 ± 4.94^b,c,A,B^	16.02 ± 2.41^b,B^	19.98 ± 1.35^a,A^	17.94 ± 5.02^a,c,A^	11.16 ± 0.84^b,A^	0.17 ± 0.02^a,A^	0.17 ± 0.02^a,A^	0.19 ± 0.02^a,A^
original	22.38 ± 6.40^a,A^	19.49 ± 0.76^c,d,A^	19.89 ± 5.07^b,A^	15.08 ± 3.76^a,A^	11.77 ± 2.43^c,A^	13.30 ± 3.76^b,A^	0.11 ± 0.02^a,A^	0.10 ± 0.01^b,c,d,A^	0.11 ± 0.02^b,c,A^

	springiness (N)			gumminess (N)			chewiness (N)		
day									
	3	7	15	3	7	15	3	7	15
BF	0.14 ± 0.01^a,A^	0.12 ± 0.02^a,A^	0.11 ± 0.01^b,c,A^	5.66 ± 0.59^a,A^	2.84 ± 0.89^b,c,d,B^	2.29 ± 0.55^b,c,d,B^	0.84 ± 0.11^a,A^	0.24 ± 0.02^a,d,B^	0.30 ± 0.08^d,B^
BF12	0.16 ± 0.01^a,A^	0.15 ± 0.01^a,A^	0.16 ± 0.01^a,A^	6.44 ± 2.54^a,A^	5.28 ± 0.58^a,b,A^	5.08 ± 1.04^a,b,A^	1.08 ± 0.54^a,A^	0.82 ± 0.14^a,A^	0.82 ± 0.14^a,A^
BF-BCFS	0.14 ± 0.02^a,b,A^	0.13 ± 0.01^a,A^	0.16 ± 0.01^a,A^	5.17 ± 1.90^a,A^	2.92 ± 0.53^a,d,A^	4.86 ± 0.61^a,c,A^	0.86 ± 0.48^a,A^	0.46 ± 0.05^a,c,A^	0.79 ± 0.07^a,b,A^
BF-BCFS12	0.14 ± 0.02^a,c,A^	0.14 ± 0.01^a,A^	0.14 ± 0.02^a,b,A^	4.70 ± 0.54^a,A^	4.25 ± 1.27^a,c,A^	3.1 ± 0.32^a,d,A^	0.70 ± 0.19^a,A^	0.61 ± 0.22^a,b,A^	0.44 ± 0.11^b,c,d,A^
original	0.09 ± 0.01^b,c,d,A^	0.06 ± 0.01^b,A^	0.07 ± 0.01^c,A^	2.62 ± 0.76^a,A^	1.98 ± 0.35^c,d,A^	2.29 ± 0.94^d,A^	0.24 ± 0.09^a,A^	0.13 ± 0.04^b,c,d,A^	0.17 ± 0.09^d,A^

a Day zero (control) for cream cracker parameters on the original packaging (N): Hardness: 36.45 ± 3.10; Strength maximum: 26.47 ± 2.26; Cohesiveness: 0.15 ± 0.01; Springiness: 0.12 ± 0.01; Gumminess: 5.56 ± 0.75; Chewiness: 0.68 ± 0.14. Different superscript letters mean statistical difference (*p* < 0.05), lowercase letters compare packages within the same checkpoint for each parameter and uppercase letters compare the same package at different checkpoints for each parameter.

It is noteworthy that the solubility values of these films were significantly lower than those of castor cake protein films modified with glutaraldehyde (solubility = 65–76%)^33^ and castor cake protein films modified with gallic acid (solubility = 76–91%).^34^ The films in this study exhibited very low water solubility (∼0.78 ± 0.20%), reflecting their hydrophobic surface, as indicated by contact angle measurements. In contrast, Maniglia et al.^7^ reported higher solubility (39.02 ± 0.31%), likely due to methodological differences. For example, in this study the alcalinization can modifies the structure of proteins, which consequently influences their solubility and show differences from other similar babassu studies. Such low solubility may benefit food applications by helping to preserve product and package integrity under high humidity.

#### 3.3.3 Water Vapor Permeability by Permeation Cell and Texture Analysis of Cream Crackers Biscuits

Alkaline treatment increases the WVP.^22^ This increase is also associated with a high concentration of hydrophilic groups and the porosity of the fibers in BM and babassu cake sieved to 100 mesh (BC100), which generate stronger interactions between water molecules and greater water permeability through the pores.^7^ However, these factors had no effect on the WVP of the films in this study. Compared to the higher WVP of babassu mesocarp films reported by Maniglia et al.^7^ (9.30 ± 0.30 × 10^–10^ g/m-s-Pa), the films in this study had lower values, averaging 3.36 ± 1.49 × 10^–10^ g/m-s-Pa, similar to those of babassu starch films. As an innovative approach, biscuit texture parameters were analyzed to establish a relationship with WVP in a real food packaging system (Table 3).

Hardness and crispness are the most critical factors in evaluating the texture of biscuits. Previous studies have reported that a decrease in hardness and an increase in chewiness are associated with a loss of crispness, which consumers find undesirable. These texture characteristics relate to a higher WVP, which facilitates the diffusion of water molecules that have passed through the packaging.^18,19,35^

The data presented in the legend of Table 3 detail the texture of the biscuits on day zero, immediately after opening the original packaging. This information was used to assess which film best preserved the texture. On day 3, no significant differences were found between the packages. On day 7, BF12 and BF-BCFS12 demonstrated superior biscuit parameters, outperforming the original and untreated wrappers. On day 15, only BF12 exhibited a texture comparable to the control.

Comparing the performance of the individual films at different checkpoints, BF demonstrated a decrease in most texture parameters between days 3 and 7, except for springiness and cohesiveness, which remained stable. No significant changes occurred between days 7 and 15. BF-BCFS12 exhibited a slight decrease in hardness on day 15, while BF12 and BF-BCFS showed no significant changes between checkpoints. Thus, BF12 preserved the texture of the biscuits better than the other packages, indicating superior performance in maintaining biscuit quality.

Crispness and hardness are key indicators of biscuit quality, according to the USDA.^36^ In this study, the alkalized films effectively preserved these texture attributes during storage, helping to maintain the biscuits’ original crunch and firmness. This preservation likely contributed to higher sensory acceptance, demonstrating the potential of the developed films for maintaining product quality. This approach focuses solely on WVP. Further analysis of shelf life is therefore required to determine the most suitable biobased packaging to replace the original.

#### 3.3.4 Colorimetric Parameters, Light Transmission, Visual Aspect, and Microstructure

The color parameters and Δ*E* values of the BF-BCFS were comparable to those of the control (BF) (Table 2), as expected since they were not subjected to chemical treatment. Minor differences may be attributed to the incorporation of BCFS, which is known to affect color.^37^ BF12 and BF-BCFS12 exhibited greater differences compared to BF, displaying the lowest L*, a*, and b* values, possibly due to the alkali treatment, which darkens the BM and BCFS^6^ by probably inducing the Maillard reaction. Heat intensifies this reaction^37^ and promotes the caramelization of sugars within the polymeric matrix.^38^ Furthermore, it is hypothesized that in this study, alkalization and heat facilitate the accelerated oxidation of phenolic compounds into quinones, resulting in the formation of brown pigments.^39^ Higher Δ*E* values were observed in the treated films, which aligns with the findings of Fai et al.,^21^ who reported Δ*E* variations attributed to treatments in biopolymer matrices. The BF12 and BF-BCFS12 samples appeared darker due to the combined effects of heat and chemical treatment, leading to lower C* values and less saturated colors compared to the naturally more vibrant BF and BF-BCFS samples. This darker pigmentation is advantageous for the development of packaging for food products requiring protection, as well as for creating anti-UV packaging.

Films that did not undergo chemical treatment exhibited low opacity and retained a degree of transparency, despite their coloration; however, this transparency was inferior to that of pure PLA films.^40^ In contrast, the treated films were darker and more opaque, limiting light transmission. Figure 1 shows the UV and visible light transmission of the four films.

**Figure 1:**
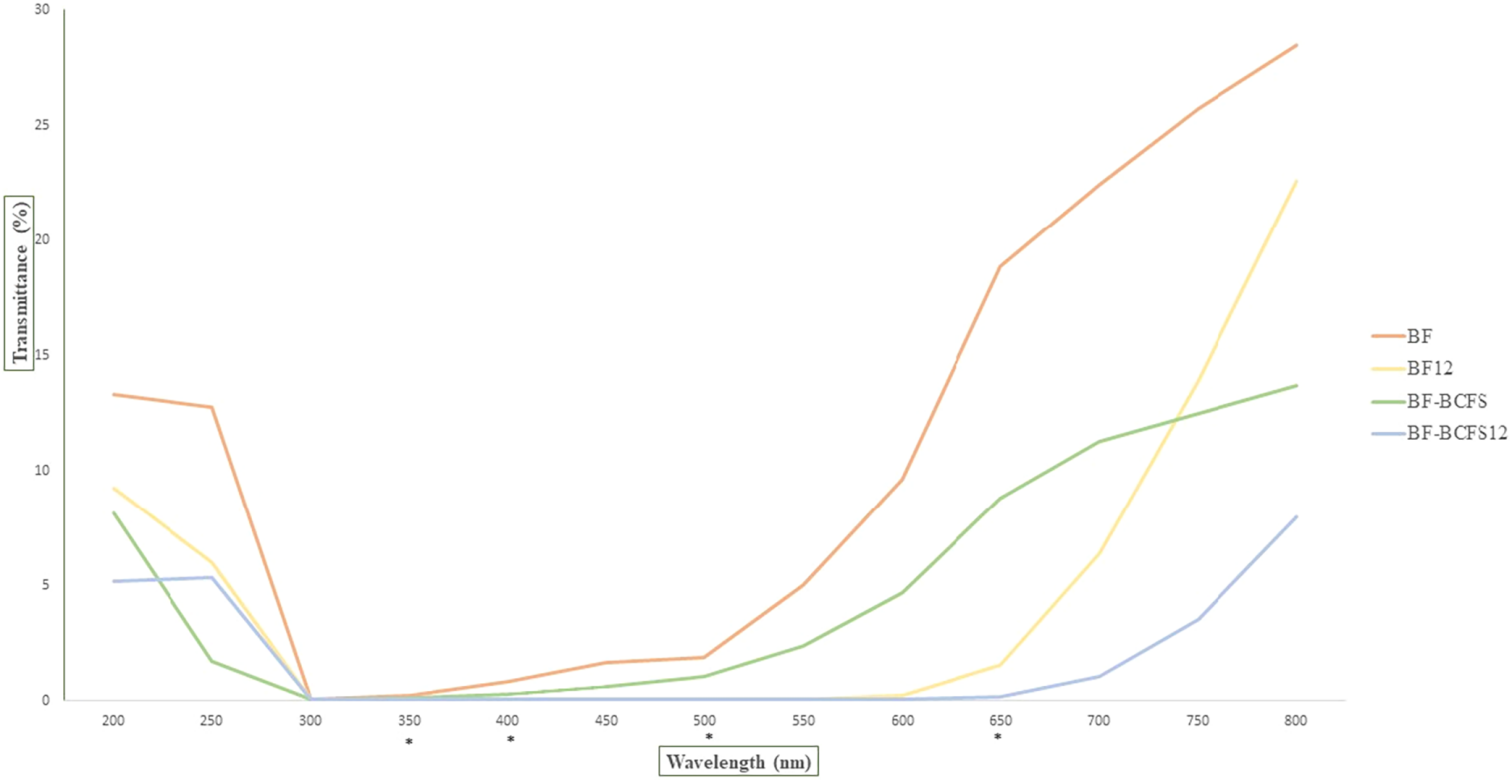
Percentual of transmittance of the BF, BF12, BF-BCFS, and BF-BCFS12 films BF: babassu-based film; BF12: babassu-based film at pH 12; BF-BCFS: babassu cake-filtered supernatant film; BF-BCFS12: babassu cake-filtered supernatant film at pH 12 (* = significant difference between any film in wavelength).

BF-BCFS differed from BF12 at 400, 500, and 650 nm, from BF-BCFS12 at 400 and 500 nm, and from BF at 650 nm. Additionally, BF-BCFS12 displayed differences compared to BF at 350 and 400 nm, and to BF12 at the same wavelengths. These variations suggest that films subjected to alkaline treatment exhibit decreased transmittance across multiple wavelengths, thereby enhancing their light barrier properties. Coupled with the inherent coloration of the films, this characteristic renders them suitable for protecting light-sensitive food products and may contribute to an extension of shelf life.

Surface and fracture morphologies are the result of molecular interactions within polymer matrices.^49^ Both BF and BF-BCFS exhibited comparable surface morphologies, as shown in Figure 2. However, BF displayed a smoother and more homogeneous surface, attributable to the fibrous portion of the BCFS and its pronounced granulometry compared to the BM, which prevents complete incorporation and leads to tiny air pores during processing, resulting in cavities and fractures that increase WVP.^6,7^ Cross-sectional analysis revealed that BF had more regular laminate structures than BF-BCFS, consistent with Maniglia et al.^7^ BF12 and BF-BCFS12 had rougher surfaces than BF and BF-BCFS, but their cross-sectional morphology was similar to that of their respective counterparts at native pH.

**Figure 2:**
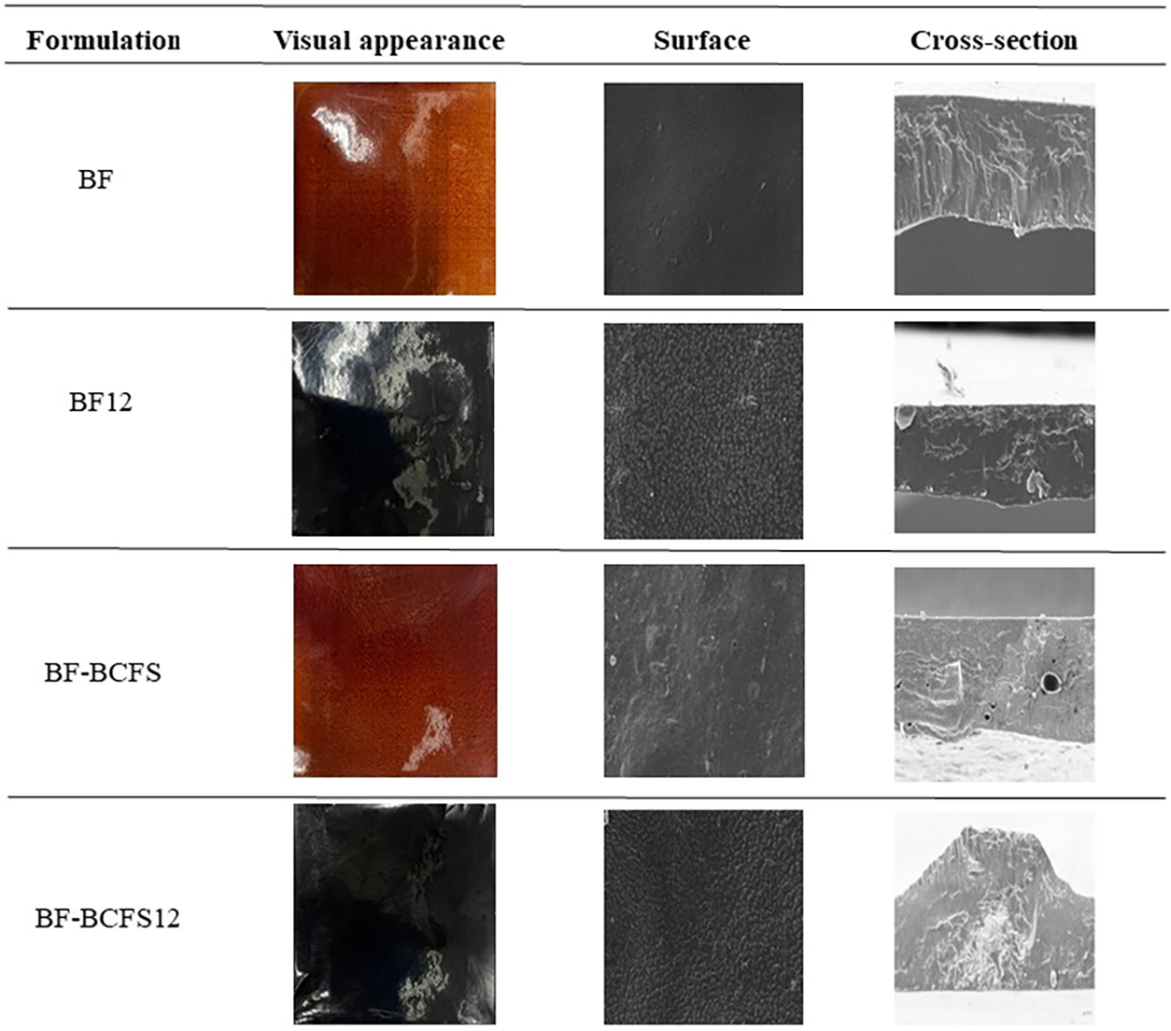
Visual appearance and SEM micrographs of the surfaces and cross sections of the BF, BF12, BF-BCFS, and BF-BCFS12 films (magnification ×1000). BF: babassu-based film; BF12: babassu-based film at pH 12; BF-BCFS: babassu cake-filtered supernatant film; BF-BCFS12: babassu cake-filtered supernatant film at pH 12.

#### 3.3.5 Chemical and Thermal Properties

In the FT-IR analysis (Figure 3) conducted within the 3250–3000 cm^–1^ range, all films displayed bands associated with O–H bonds.^13,6^ Notable differences among the formulations were evident in this spectral region. The BF film exhibited a sharper and more intense band, indicative of a higher concentration of free hydroxyl groups and diminished intermolecular interactions. Following alkaline treatment (BF12), this band broadened and experienced a slight shift to lower wavenumbers, suggesting an enhancement in hydrogen bonding and a rearrangement of the polymeric chains. The incorporation of BCFS (BF–BCFS) resulted in decreased band intensity and slight broadening, signifying the formation of intermolecular hydrogen bonds between phenolic compounds from BCFS and the starch-based matrix. In the BF–BCFS12 formulation, the O–H band was the broadest and most displaced, aligning with the formation of stronger and more heterogeneous hydrogen bonds. These findings substantiate the hypothesis of enhanced molecular interactions within the polymer network, which are directly correlated with increased structural cohesion, thermal stability, and improved mechanical and barrier properties, as discussed in other sections. The 2900–2750 cm^–1^ band corresponds to the methoxyl group (CH3O−) and C–H bonds associated with proteins.^6^ These bands were more pronounced, especially in BF-BCFS12, indicating molecular rearrangement and enhanced cross-linking between starch and protein fractions. The spectral range between 1750 and 1500 cm^–1^ indicates the presence of C=O bonds associated with the amide group of proteins and the ester group in triglycerides, as well as the amide II band generated by vibrations between N–H and C–N.^13^ This band became more intense and experienced a slight shift in BF-BCFS12, suggesting the formation of new hydrogen bonds and potential partial esterification or complexation between polysaccharides and protein–lipid residues. Such bonds are also detectable in the 1250 cm^–1^ range, particularly in alkaline films. The band between 1100 and 900 cm^–1^ corresponds to C–C, C–O, and C–H bonds,^13^ which are characteristic of saccharides. It is more intense in the BF and BF12 formulations, indicating a higher degree of crystallinity, which was partially disrupted by the incorporation of BCFS and alkaline modification. Collectively, these spectral changes provide evidence for the occurrence of new intermolecular interactions that modulate both the molecular packing and crystallinity of the films.

**Figure 3:**
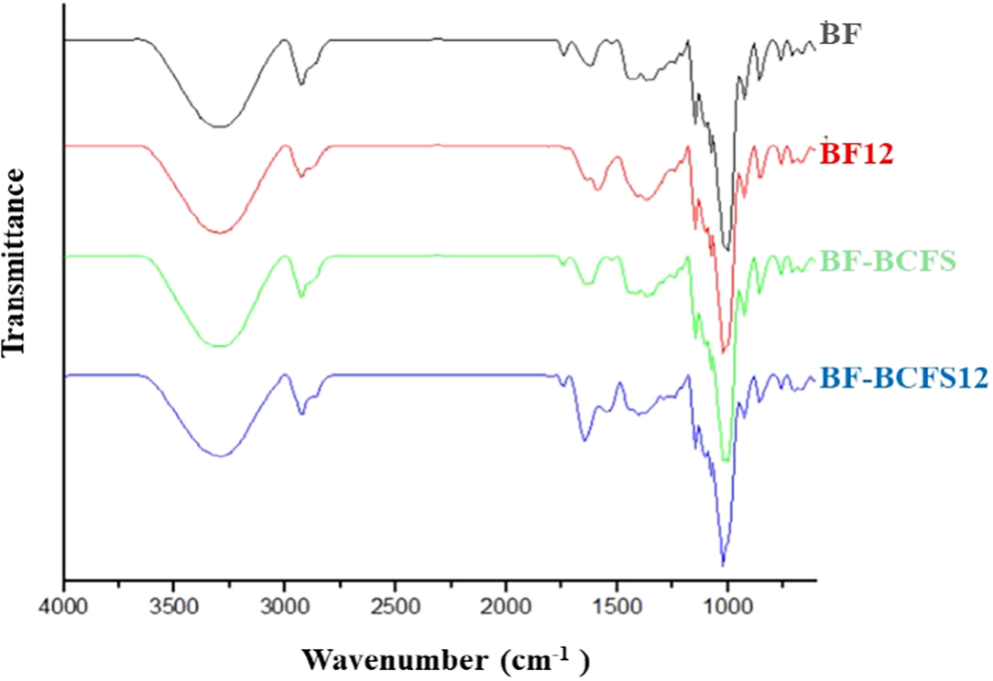
FT-IR spectral patterns of the BF, BF12, BF-BCFS, and BF-BCFS12 films. BF: babassu-based film; BF12: babassu-based film at pH 12; BF-BCFS: babassu cake-filtered supernatant film; BF-BCFS12: babassu cake-filtered supernatant film at pH 12.

In thermal analysis (Figure 4), the nontreated films, BF and BF-BCFS, exhibited the lowest glass transition temperature (*T*_g_) values (65.18 and 69.15 °C), associated with the transition from a brittle to a viscous material.^41^ The enthalpy (Δ*H*) for these samples was 195.91 and 232.1 J/g, respectively (Figure 5). Comparing these results suggests that the addition of BCFS strengthens bonds between polymer components, requiring more energy for the glass transition.^41^ The alkaline treatment effectively improved thermal resistance and the energy required for the transition (*T*_g_: 76.28 °C and Δ*H*: 325.5 J/g) of a matrix with previously weak bonds, as observed for BF. The BF-BCFS12 formulation exhibited three Tg points, with the second and third transitions occurring at higher temperatures than the other formulations. This increased number of Tg points is hypothesized to result from different compounds whose interactions were modified by the addition of BCFS and its chemical treatment. The first degradation event occurred within the temperature range of 57 to 71 °C, related to water evaporation.^6^ The treated films, BF12 and BF-BCFS12, showed degradation at higher temperatures (∼71 °C) with mass losses of 10.50% and 9.53%, respectively. A second event for BF and BF-BCFS occurred at 149 and 137 °C, accompanied by a mass loss of about 4%. The third event, occurring at 207 °C, was associated with a loss of 7.84% and 9.45%, indicating degradation of the plasticizing compounds.^31^ The fourth event, occurring at temperatures of 292 and 305 °C, resulted in a mass loss of 43.79% for BF and 46.21% for BF-BCFS, associated with starch degradation.^42^ Glycerol reaches maximum volatility at about 237 °C.^6^ In the range of 200–250 °C, saccharide ring degradation begins.^42^ These circumstances relate to the second BF12 event at 236 °C, where a mass loss of 51.62% occurs. Starch degradation occurs between 270 and 450 °C,^42^ explaining the second event at 281 °C and the greater mass loss (51.62%) of BF-BCFS12. The third event in the same production occurred at 467 °C, resulting in a loss of 33.13% and is related to the degradation of lignocellulosic compounds, as demonstrated by De Farias et al. (2021).^6^

**Figure 4:**
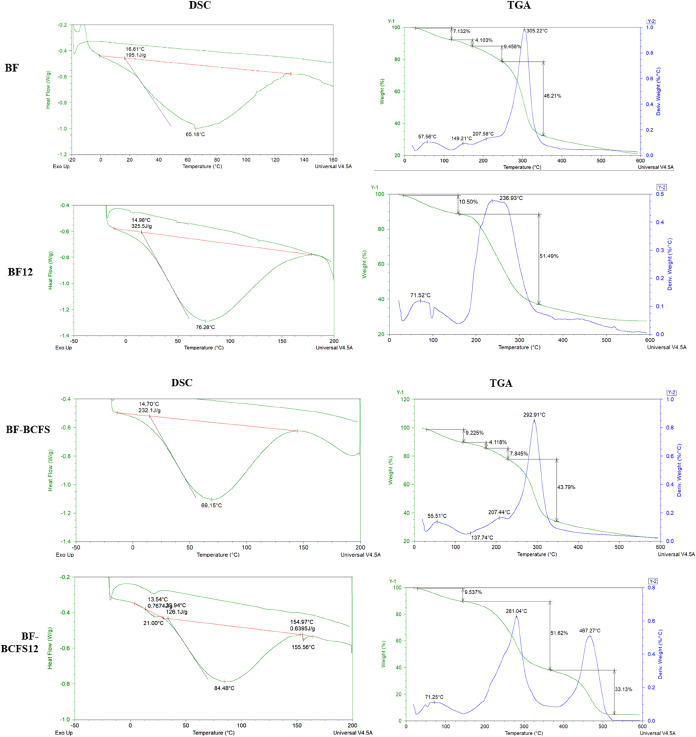
Thermal analysis (DSC and TGA) of BF, BF12, BF-BCFS, and BF-BCFS12 films. BF: babassu-based film; BF12: babassu-based film at pH 12; BF-BCFS: babassu cake-filtered supernatant film; BF-BCFS12: babassu cake-filtered supernatant film at pH 12.

**Figure 5:**
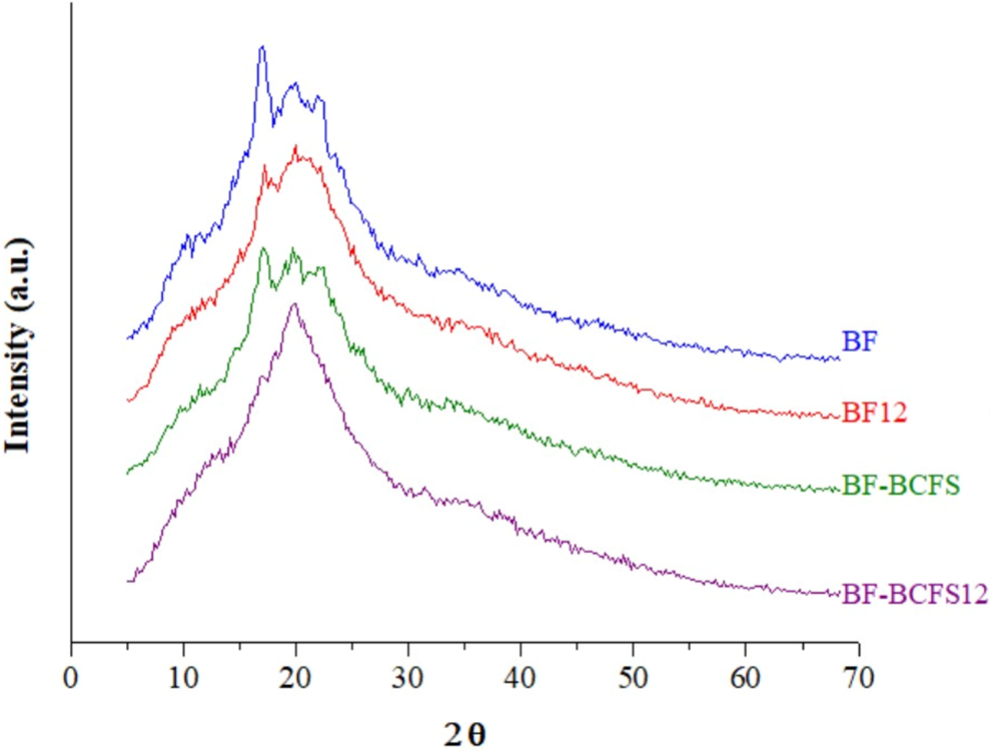
Crystallinity indices of BF, BF12, BF-BCFS, and BF-BCFS12 films Values with different letters are significantly different according to Tukey’s test (*p* < 0.05). BF: babassu-based film; BF12: babassu-based film at pH 12; BF-BCFS: babassu cake-filtered supernatant film; BF-BCFS12: babassu cake-filtered supernatant film at pH 12.

In general, the addition of BCFS and alkaline treatment led to higher *T*_g_ and Δ*H* values, indicating stronger molecular interactions and a more compact polymer network that demands greater energy for structural rearrangement. In BF-BCFS12, the presence of multiple Tg points suggests different polymeric domains formed by starch–protein–lipid associations, which may explain its greater resistance at break, rigidity, and better barrier cohesion.

Films treated under alkaline conditions (BF12 and BF-BCFS12) also showed degradation at higher temperatures, confirming improved thermal resistance and stability. These effects are likely related to the partial deprotonation of hydroxyl groups, promoting new hydrogen-bonding interactions between starch and BCFS components, as observed in FT-IR results. Consequently, the films become mechanically stronger and less permeable, with a more ordered and stable structure. Together, the FT-IR and thermal analyses demonstrate that the chemical modifications introduced by BCFS incorporation and alkaline treatment are directly reflected in the improved macroscopic performance of the films.

#### 3.3.6 Crystallinity Index

Figure 5 presents the crystallinity indices (%) for BF, BF12, BF-BCFS, and BF-BCFS12 as 7.35 ± 0.09, 4.50 ± 0.04, 5.72 ± 0.06, and 3.21 ± 0.10, respectively, with significant differences among them. Films containing BM (BF and BF12) exhibited more pronounced crystalline regions than those with BCFS, likely due to the higher content of fibers, proteins, and lipids in the latter, which can hinder amylose retrogradation and interfere with optimal structural bonding within the polymeric matrix.^6^ BF12 and BF-BCFS12 displayed reduced crystallinity, indicating that alkaline treatment may induce molecular disorder, leading to a more amorphous structure that enhances flexibility and alters thermal behavior.^41^

#### 3.3.7 Surface Wettability

The contact angles for water and oil (Figure 6) showed that BF and BF-BCFS exhibited, on average, moderate hydrophilicity (∼59 ± 5°), comparable to the babassu mesocarp films (56 ± 1°).^7^ Alkaline treatment decreased the hydrophilicity observed in BF12 and BF-BCFS12 (∼73 ± 5°), probably due to increased lipid content as seen in BCFS12 (Supporting Information). All films were lipophilic in oil (<17 ± 5°), a property attributed to the presence of nonpolar components such as lignin, phenolic compounds and fats in the BF and BF-BCFS films,^43,44^ as well as to the migration of lipids to the surface after chemical treatment.^7^

**Figure 6:**
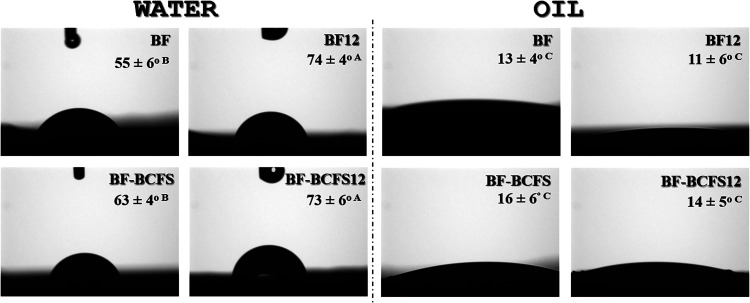
Contact angle images the BF, BF12, BF-BCFS, and BF-BCFS12 films with the respective contact angle values for water and oil drop Values with different letters are significantly different according to Tukey’s test (*p* < 0.05) BF: babassu-based film; BF12: babassu-based film at pH 12; BF-BCFS: babassu cake-filtered supernatant film; BF-BCFS12: babassu cake-filtered supernatant film at pH 12.

#### 3.3.8 Heat Seal Strength

All films could be thermally sealed. Prolonged heating above 70 °C for five seconds caused all films to lose integrity during sealing. Before this threshold, disentanglement of the polymer molecules led to slight openings at the sealing interface (peeling mode).^45^

The seal strength did not significantly differ among the films (BF = 0.005 ± 0.02 N/mm; BF12 = 0.009 ± 0.02 N/mm; BF-BCFS = 0.007 ± 0.01 N/mm; BF-BCFS12 = 0.008 ± 0.02 N/mm), although these values were lower compared to other biobased films.^45,46^ However, low seal strength can be advantageous for consumer convenience, facilitating easier opening, particularly in single-use products such as sauces and oils.

### 3.4 Film Application as a Single-Use Flexible Sachet for Food Packaging

#### 3.4.1 Stability Tests of Soybean and Virgin Olive Oil in the Sachet

BF-BCFS12 was selected for its anti-UV properties for packaging soybean and olive oil to evaluate its effectiveness in preserving oils under oxidative conditions (Table 4).

**Table 4:**
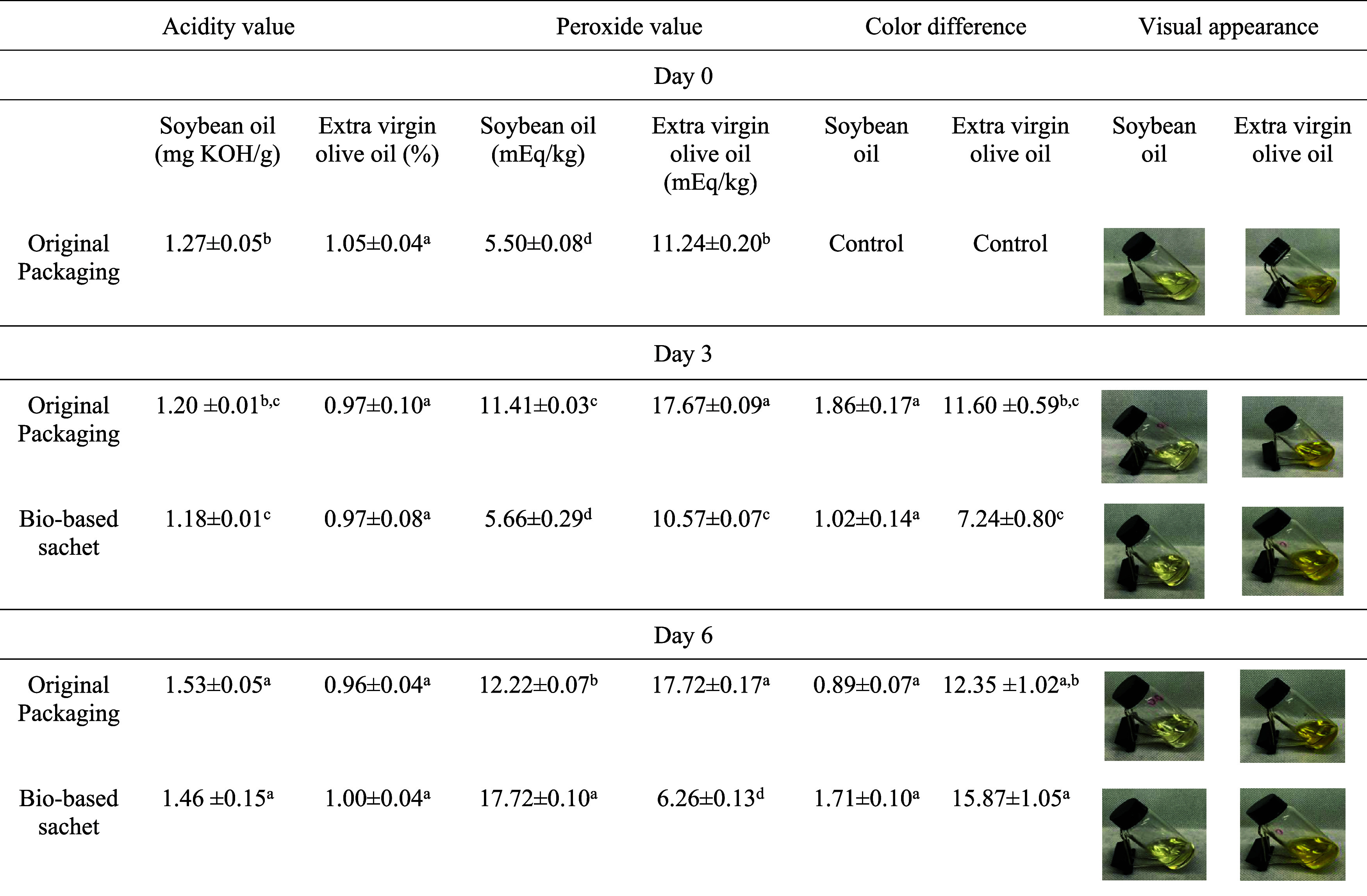
Oxidation Parameters of Soybean Oil and Extra Virgin Olive Oil in Original and Bio-Based Packaging^a^

a Values with different letters in the same column are significantly different according to Tukey’s test (*p* < 0.05).

The commercial soybean oil had an acidity of 1.27 ± 0.05 mg KOH/g on day zero, while the olive oil had an acidity of 1.05 ± 0.04%. Both exceeded the permissible limit, which is ≤ 0.20 mg KOH/g for refined vegetable oils^47^ and ≤0.8% for olive oil.^48^ The peroxide content of soybean oil was 5.50 ± 0.08 mEq/kg, also above the legal quality limit (≤2.5 mEq/kg).^47^ These altered values indicate possible oxidation, even in conventional packaging, suggesting that some Brazilian commercial oils may not meet quality standards. On day 3, the acidity of soybean oil remained stable in the original packaging (1.20 ± 0.01 mg KOH/g), whereas the sachet showed a slight reduction to 1.18 ± 0.01 mg KOH/g. By day 6, the acidity in both packages increased compared to day zero. No significant differences were observed between packaging types regarding the acidity of soybean oil. For olive oil, acidity remained stable on days 0, 3, and 6, with no significant differences between packaging types. These results suggest that the sachet preserves acidity similarly to the original packaging.

When stored in original packaging, the peroxide value of soybean oil increased significantly from day 0 (5.50 ± 0.00 mEq/kg) to day 3 (11.41 ± 0.03 mEq/kg), then slowed until day 6 (12.22 ± 0.07 mEq/kg). In contrast, the peroxide content in the sachet remained stable until day 3 (5.66 ± 0.29 mEq/kg) before rising significantly until day 6 (17.72 ± 0.1 mEq/kg). This indicates accelerated oxidation at later stages beyond levels observed in original packaging.^49^

On day 0, olive oil complied with Brazilian legislation for the peroxide limit (≤20 mEq/kg),^50^ but on day 3 both packages exceeded this value. The original packaging showed a significant increase from day 0, stabilizing by day 6 (17.72 ± 0.17 mEq/kg). In contrast, the sachet decreased from day 3 (10.57 ± 0.07 mEq/kg) to day 6 (6.26 ± 0.13 mEq/kg), indicating advanced oxidation and the formation of secondary compounds.^50^ The fatty acid composition of olive oil, characterized by a high content of monounsaturated fatty acids (MUFA) and a low content of polyunsaturated fatty acids (PUFA), along with a reduced number of bis-allylic positions, contributes to its enhanced stability against oxidation. Nevertheless, under pro-oxidative conditions, such as exposure to heat and light, the bioactive compounds present in olive oil exhibit greater susceptibility to degradation compared to the tocopherols found in soybean oil, thereby elucidating the observed phenomena.^51,52^

The appearance of the oils is shown in Table 4. There were no differences between the packaging of the two oils at the same checkpoints. However, the olive oil in original packaging exhibited better color retention, with color stability remaining similar at day 3 (Δ = 11.6 ± 3.91) and day 6 (Δ = 12.35 ± 1.02).

The results suggest that sachets have the potential to serve as single-use alternatives for oil preservation, despite the possibility of a higher dissolved oxygen content compared to conventional packaging. This effect is attributed to the greater surface area-to-volume ratio present in smaller packages, improving contact between the product and oxygen, which can promote oxidative reactions. Further studies are needed to determine the extent to which these factors influence the oxidative stability of oils in such packaging. The excellent preservation of the oils in the sachets, especially soybean oil, demonstrates that this biobased packaging can protect against oxidation as effectively as conventional packaging. This was also reported by Dong et al.,^53^ and Chavoshizadeh et al.^54^

#### 3.4.2 Evaluation of the Performance of Bio-Based Sachet During Storage of Soybean and Virgin Olive Oil

Figure 7 illustrates the development of maximum strength of the sachets during the storage of the oils. Higher temperatures cause molecular chains to break and reduce moisture, leading to water loss in glycerol molecules. This reduces flexibility and strength, which declines with increased exposure to heat.^55,56^

**Figure 7:**
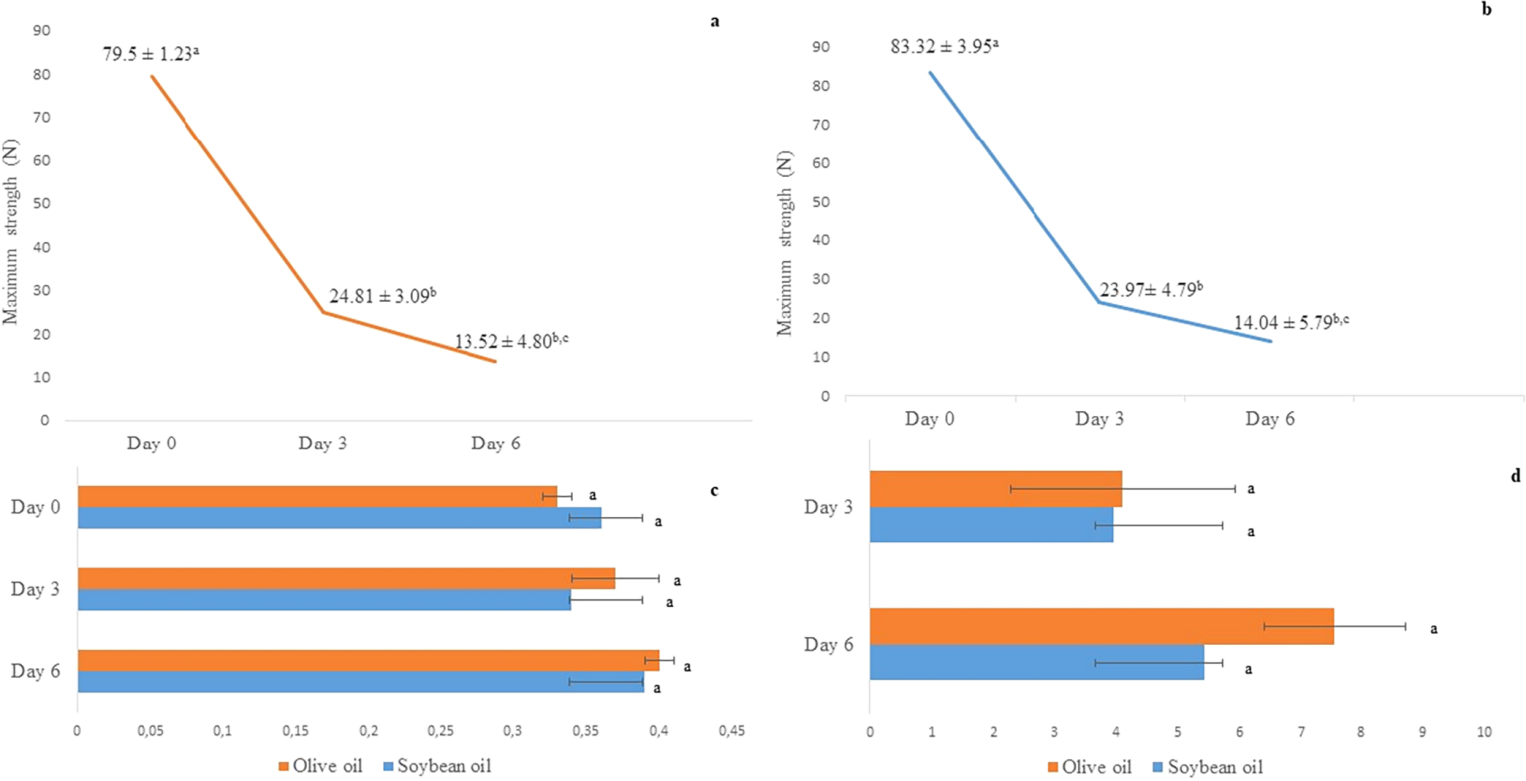
Maximum strength (N) and visual aspect of olive (a) and soybean oil (b) sachets, thickness (c), and color differences of sachets (d) Values with different letters are significantly different according to Tukey’s test (*p* < 0.05).

The tensile strength of the sachets decreased considerably for both oils over time. For soybean oil, values dropped from 83.32 N on day 0 to 23.97 N on day 3 (−71.2%) and 14.04 N on day 6 (−41.5% compared to day 3). Similarly, olive oil sachets decreased from 79.50 to 24.81 N (−68.8%) and 13.52 N (−45.5%) over the same period. These decreases indicate material degradation with prolonged exposure to elevated temperatures, consistent with results observed by Zerihun et al.^57^

The thickness of the sachets for soybean and olive oil at the three checkpoints was 0.36 ± 0.01; 0.34 ± 0.03; 0.39 ± 0.01 mm and 0.33 ± 0.01; 0.37 ± 0.04; 0.40 ± 0.02 mm. The color differences of the sachets with soybean and olive oil were 3.97 ± 1.82 and 4.11 ± 1.47 on day 3 and 5.43 ± 1.15 and 7.56 ± 0.80 on day 6, respectively. No significant differences were found for either parameter at the different checkpoints. The most noticeable visual change in the sachet was increased roughness due to heat exposure and water loss.^58^ Nevertheless, the BF-BCFS12 sachet proved to be a robust single-use packaging for both oils, even under accelerated storage conditions.

## 4 Conclusions

Alkaline treatment enhanced the functionality of babassu-based films. Additionally, the integration of BCFS, a lipid-rich and upcycled byproduct, significantly augmented their performance. The synergistic interaction between alkali treatment and BCFS incorporation resulted in the development of BF-BCFS12, which demonstrated superior thermal resistance, mechanical strength, UV-blocking capabilities, and oxidative protection, rendering it suitable for use as sachets for oily foods. These findings position BCFS as an innovative raw material that not only valorizes an agro-industrial byproduct but also enables the production of water-insoluble, biobased films suitable for the active packaging of oxidation-sensitive foods, such as butter, vegetable oils, oilseeds, fatty meats, and sauces. While this preliminary study on the application of babassu cake in biobased films yields promising results pertinent to alternative food packaging, further research is imperative. Future investigations will focus on evaluating additional critical properties, including color stability, compound migration, antimicrobial activity, biodegradation behavior, phytotoxicity, life cycle assessment, and socio-economic impact, to facilitate potential regulatory approval and enhance commercial scalability.

## Supplementary Material



## Notes

Characterization of babassu cake, encompassing centesimal composition, colorimetric parameters, and multielement analysis; detailed description of the preparation of film-forming suspensions using hydrothermal treatment and the subsequent film development by casting (preliminary tests) (PDF)
