# Supplementary material for: Water-Insoluble Films from Upcycled Babassu Coconut Byproducts: An Alternative Material for Single-Use Oil Sachets
Source: ACS Omega. 2025 Dec 29;11(1):967–79. doi: 10.1021/acsomega.5c07743 (PMC12809288; doi:10.1021/acsomega.5c07743)
Supplement: Supplementary file 1 [file ao5c07743_si_001.pdf]

Supporting Information

Water-insoluble films from upcycled babassu coconut by-products: An alternative material for single-use oil sachets

Letícia de Oliveira Gonçalves[1]; Patrícia Marques De Farias [2]; Yan Fonseca dos Santos[3]; Jefferson Santos de Gois[4]; Bianca Chieregato Maniglia[5]; Ana Elizabeth Cavalcante Fai[1,3]*

[1] Food and Nutrition Graduate Program, Federal University of the State of Rio de Janeiro - UNIRIO, Av. Pasteur, 296, Urca, Rio de Janeiro/RJ, 22290-240, Brazil; nutricionistaleticiagoncalves@gmail.com

[2] Sustainable Packaging Institute SPI, Faculty of Life Sciences, Albstadt-Sigmaringen University, Anton-Günther-Str. 51, Sigmaringen 72488, Germany; patriciamfarias@hotmail.com

[3] Laboratory of Multidisciplinary Practices for Sustainability (LAMPS), Institute of Nutrition, State University of Rio de Janeiro - UERJ, R. São Francisco Xavier, 524, Maracanã, Rio de Janeiro/RJ, 20550-013, Brazil; yanfsan@gmail.com

[4] Department of Analytical Chemistry, Rio de Janeiro State University (UERJ), Rio de Janeiro, RJ, Brazil; jeffersonsgois@gmail.com

[5] São Carlos Institute of Chemistry, University of São Paulo – USP, Av. Trabalhador São-Carlense, São Carlos, SP, Brazil; biancamaniglia@iqsc.usp.br

*Corresponding author: Ana Elizabeth Cavalcante Fai, e-mail: ana.fai@uerj.br

1. Methods

1.1 Preparation of film-forming suspensions using hydrothermal treatment and film development by casting - Preliminary tests

Preliminary tests were undertaken to establish the methodology for the film-forming suspension (FFS):

(I) Babassu cake flour (both sieved and unsieved) was combined with water and glycerol, subsequently heated in a water bath;

(II) (II) Babassu cake flour (sieved and unsieved) was combined with water and glycerol, subjected to heating in both an autoclave and a water bath;

(III) (III) Babassu cake supernatant was analyzed at various pH (ranging from 1 to 12) in combination with glycerol, and heated in both an autoclave and a water bath;

(IV) (IV) Babassu cake supernatant was mixed with babassu mesocarp flour (BM) at varying pH (1 to 12) and heated in a water bath; and

(V) (V) Babassu cake supernatant was combined with BM at pH levels of 6 and 12, subjected to heating in both an autoclave and a water bath.

The findings from Experiments I, II, and III did not demonstrate successful film formation. In Experiment IV, it was noted that the film-forming suspensions produced cohesive films exhibiting a distinct color at pH 12. De Farias et al. (2023) [4] similarly reported that films within this pH range exhibited increased amorphous characteristics, which enhance chain mobility and elongation at break, ultimately reducing brittleness. Therefore, the focus was directed towards pH values of 6 and 12, with pH 6 designated as the native pH (control). Accordingly, the preparation for Experiment V was tested exclusively at these two pH values, resulting in the formation of homogeneous and cohesive films without cracks. In response to these challenges, BC was processed using

an adapted methodology from De Farias et al. (2023) [4]. The BC was ground in a Philips Walita (Brazil) machine and sieved to a mesh size of 100. Two 5% (w/w) BC100 suspensions were prepared: one using distilled water (native pH ~6) and the other with 1 M NaOH to achieve a pH of 12 (1 mL NaOH per 20 g of supernatant). Following a cooling period of 12 hours, the suspensions were filtered through a cloth. The resulting supernatants, designated BCFS (pH ~6) and BCFS12 (pH 12), contained 2.5 g/L and 10 g/L of soluble solids, respectively, and were utilized to prepare the FFS. The centesimal compositions were determined as outlined in Section 2.2.

2. Results and Discussion

2.1 Characterization of Babassu Cake: Chemical Composition, Colorimetric Parameters, and Multielement Analysis

The centesimal composition correlates with the colorimetric parameters (Table 1). BC100 exhibited a higher protein and lipid content and presented a darker, more intense orange color than BC. This variation may be attributed to sieving, which involves mechanical agitation and promotes the release of colored compounds, including proteins, lipids, metals, hydrophobic pigments, and phenols [7,8]. The released phenols directly influence the total phenolic and polyphenolic content, which was higher in BC100 than in BC. Babassu cake contained approximately twice the total phenolic and polyphenolic content compared to blackberry cake (49.80 µg/g and 96.46 mg/100 g, respectively), a fruit recognized for its bioactive compounds [9] (Table 1). These results underscore the potential of babassu cake as a bioactive raw material for the development of antioxidant bio-based films.

The centesimal composition of BC is comparable to that reported by Ferrari and Soler (2015) [10], who found a higher lipid content (28.8%) and a lower protein content (18.8%). Both BC and BC100 compositions are suitable for bio-based films, as the

combination of proteins and lipids can enhance mechanical properties while reducing moisture and water vapor permeability [11].

Lopes et al. (2016) [12] reported the presence of aluminum, manganese, copper, and zinc in babassu kernel at levels of 12.4 ± 0.04, 32.2 ± 1.2, 14.1 ± 0.6, and 20.1 ± 1.1 g/100 g, respectively. As shown in Table 2, BC and BC100 exhibited higher concentrations of these elements compared to whole kernels, emphasizing the added value of this by-product. The composition of micronutrients in BC and BC100 is significant from a compostable packaging perspective, as these micronutrients can enrich the soil during biodegradation and promote and accelerate seed germination [13].

Table 1. Centesimal composition and color parameters of the whole (BC), ≤100 mesh babassu cake (BC100), BF-BCFS and BC-BCFS12.

Sample	Centesimal composition (%)					Color parameters			
	Ashes	Moisture	Lipid	Protein	Carbohydrate	L*	a*	b*	Chroma value (C*)
BC	3.89±0.04[a,b]	8.52±0.20[c]	13.10±0.16[b]	21.83±0.66[b]	52.66±0.64[a]	62.48±0.88[a]	6.71±0.12[b]	15.44±0.34[b]	16.84±0.35[b]
BC100	4.16±0.02[a]	7.07±0.06[d]	15.24±0.13[a]	24.73±0.50[a]	48.81±0.46[b]	52.01±0.26[b]	8.15±0.16[a]	16.52±0.32[a]	18.42±0.36[a]
BF-BCFS	1.19±0.05[b,c]	44.91±0.25[a]	0.12±0.12[d]	15.50±2.07[d]	38.38±2.34[d]	-	-	-	-
BF-BCFS12	0.64±0.04[c]	31.71±0.48[b]	3.08±0.26[c]	19.19±0.56[b,c]	45.39±0.27[c]	-	-	-	-

BC: babassu cake; BC100: sieved babassu cake; BF-BCFS: babassu cake-filtered supernatant film; BF-BCFS12: babassu cake-filtered supernatant film at pH 12. Values with different letters in the same column are significantly different according to Tukey's test ($p < 0.05$).

Table 2. Multi-element analysis of whole (BC) and ≤100 mesh babassu cake (BC100).

Sample	Protocatechuic acid (µg/g)	Total polyphenols (mg of gallic acid /100 g)	Metals (µg/g)									
			Al	Ca	Cu	Fe	K	Mg	Mn	Na	P	Zn
BC	112±1[b]	226±9[b]	12.5±1.7[b]	864±23[b]	29.6±0.2[a]	122±16[b]	9780±231[a]	4021±95[b]	275±7[b]	50.6±1.5[a]	19492±502[b]	69.3±2.0[b]
BC100	114±6[a]	253±10[a]	19.3±0.4[a]	905±14[a]	30.5±0.5[a]	170±6[a]	9707±67[b]	4060±54[a]	322±11[a]	50.7±0.7[a]	19859±233[a]	78.1±2.5[a]

BC: babassu cake; BC100: sieved babassu cake

Al: Aluminum; Ca: Calcium; Cu: Copper; Fe: Iron; K: Potassium; Mg: Magnesium; Mn: Manganese; Na: Sodium; P: Phosphorus; Zn: Zinc.

Values with different letters in the same column are significantly different according to Tukey's test ($p < 0.05$).

2.2 Chemical Composition of Babassu Cake-Filtered Supernatants

The centesimal composition of the supernatants (Table 1) demonstrates that alkaline treatment (pH 12) is more effective in solubilizing lipids, proteins, and fibers compared to treatment with water, thereby rendering these compounds suitable for the formation of a film matrix. Alkaline hydrolysis facilitates the solubilization of lipids through saponification, resulting in the production of glycerol and fatty acid salts [14]. Moreover, this process modifies the structure of proteins, which consequently influences their solubility [15]. Additionally, alkalinization enhances biomass and disrupts lignin bonds, thereby releasing carbohydrates that become more accessible for hydrolysis into simpler sugars [16].

References

1. *Instituto Adolfo Lutz (IAL).* Métodos Físico-Químicos para Análise de Alimentos, 4ª ed.; Instituto Adolfo Lutz: São Paulo, **2008**.
2. Fai, A. E. C.; Alves de Souza, M. R.; de Barros, S. T.; Bruno, N. V.; Ferreira, M. S. L.; Gonçalves, T. C. B. D. A.; Branco de Andrade, É. C. Development and Evaluation of Biodegradable Films and Coatings Obtained from Fruit and Vegetable Residues Applied to Fresh-Cut Carrot (Daucus Carota L.). *Postharvest Biol. Technol.* **2016**, *112*, 194–204. https://doi.org/10.1016/j.postharvbio.2015.09.021
3. Gomes, T. M.; Toaldo, I. M.; Haas, I. C. da S.; Burin, V. M.; Caliari, V.; Luna, A. S.; de Gois, J. S.; Bordignon-Luiz, M. T. Differential Contribution of Grape Peel, Pulp, and Seed to Bioaccessibility of Micronutrients and Major Polyphenolic Compounds of Red and White Grapes through Simulated Human Digestion. *J. Funct. Foods* **2019**, *52* (November 2018), 699–708. https://doi.org/10.1016/j.jff.2018.11.051.
4. De Farias, P. M.; Vasconcelos, L. B.; Ferreira, M. E. S.; Alves Filho, E. G.; Tapia Blácido, D. R. Use of Chemically Treated Nopal Cladodes as Additive in the Cassava Starch Composite Films. *J. Vinyl Addit. Technol.* **2023**, *29* (6), 1109–1124. https://doi.org/10.1002/vnl.22040
5. Samir, A.; Ashour, F. H.; Hakim, A. A. A.; Bassyouni, M. Recent Advances in Biodegradable Polymers for Sustainable Applications. *Nature* **2022**, *6* (1). https://doi.org/10.1038/s41529-022-00277-7.
6. Ying, W.; Shi, Z.; Yang, H.; Xu, G.; Zheng, Z.; Yang, J. Effect of Alkaline Lignin Modification on Cellulase-Lignin Interactions and Enzymatic Saccharification Yield. *Biotechnol. Biofuels* **2018**, *11* (1), 1–13. https://doi.org/10.1186/s13068-018-1217-6.

7. Almeida, M. J. de O.; Wanderley, B. R. da S. M.; de Francisco, A.; Amante, E. R.; Fritzen Freire, C. B.; Helm, C. V.; Amboni, R. D. de M. C. Effects of Particle Size on the Physical, Chemical, and Technological Properties of Pre-Gelatinized Whole Pinhão (Araucaria Angustifolia) Flour. *J. Food Meas. Charact.* **2024**, *5709*, 5695–5709. https://doi.org/10.1007/s11694-024-02599-3.
8. Sakhare, S. D.; Inamdar, A. A.; Soumya, C.; Indrani, D.; Rao, G. V. Effect of Flour Particle Size on Microstructural, Rheological and Physico-Sensory Characteristics of Bread and South Indian Parotta. *J. Food Sci. Technol.* **2014**, *51* (12), 4108–4113. https://doi.org/10.1007/s13197-013-0939-5.
9. Miljanić, J.; Krstović, S.; Perović, L.; Kojić, J.; Travičić, V.; Bajac, B. Assessment of the Nutritional Benefits and Aflatoxin B1 Adsorption Properties of Blackberry Seed Cold-Pressed Oil By-Product. *Foods* **2024**, *13* (19). https://doi.org/10.3390/foods13193140.
10. Ferrari, R. A.; Soler, M. P. Obtention and Characterization of Coconut Babassu Derivatives. *Sci. Agric.* **2015**, *72* (4), 291–296. https://doi.org/10.1590/0103-9016-2014-0278.
11. Purewal, S. S.; Kaur, A.; Bangar, S. P.; Singh, P.; Singh, H. Protein-Based Films and Coatings: An Innovative Approach. *Coatings* **2024**, *14* (1), 1–24. https://doi.org/10.3390/coatings14010032.
12. Lopes, G. S.; Silva, F. L. F.; Grinberg, A. P.; Sturgeon, R. E. An Evaluation of the Use of Formic Acid for Extraction of Trace Elements from Brazil Nut and Babassu Coconut and Its Suitability for Multi-Element Determination by ICP-MS. *J. Braz. Chem. Soc.* **2016**, *27* (7), 1229–1235. https://doi.org/10.5935/0103-5053.20160018.
13. Tripathi, D. K.; Singh, V. P.; Chauhan, D. K.; Prasad, S. M.; Dubey, N. K. Role of Macronutrients in Plant Growth and Acclimation: Recent Advances and Future Prospects. In *Improving Crop Productivity in Sustainable Agriculture*; Tripathi, D. K., Ed.; Springer: Cham, 2014; pp 1–368. https://doi.org/10.1007/978-1-4614-8824-8
14. Maniglia, B. C.; Tessaro, L.; Lucas, A. A.; Tapia-Blácido, D. R. Bioactive Films Based on Babassu Mesocarp Flour and Starch. *Food Hydrocoll.* **2017**, *70*, 383–391. https://doi.org/10.1016/j.foodhyd.2017.04.022.
15. Momen, S.; Alavi, F.; Aider, M. Alkali-Mediated Treatments for Extraction and Functional Modification of Proteins: Critical and Application Review. *Trends Food Sci. Technol.* **2021**, *110* (February), 778–797. https://doi.org/10.1016/j.tifs.2021.02.052.
16. Worku, L. A.; Bachheti, R. K.; Bachheti, A.; Milessi, T. S.; Chandel, A. K. *Understanding the Biochemical Changes at Molecular Level during Biomass Pretreatment: A Comprehensive Analysis*; Springer Netherlands, **2024**; Vol. 31. https://doi.org/10.1007/s10570-024-06081-7
